# Intragenic *CFTR* Duplication and 5T/12TG Variant in a Patient with Non-Classic Cystic Fibrosis

**DOI:** 10.1038/srep38776

**Published:** 2016-12-20

**Authors:** Patricia B. S. Celestino-Soper, Edward Simpson, Danika Tumbleson Brink, Ty C. Lynnes, Stephen Dlouhy, Matteo Vatta, Jana Yeley, Cynthia Brown, Shaochun Bai

**Affiliations:** 1 Department of Medical and Molecular Genetics, Indiana University School of Medicine, Indianapolis, IN, 46202, USA; 2 Krannert Institute of Cardiology, Division of Cardiology, Department of Medicine, Indiana University School of Medicine, Indianapolis, IN, 46202, USA; 3 Division of Pulmonary, Allergy, Critical Care, Occupational, and Sleep Medicine, Indiana University, Indianapolis, IN, 46202, USA

## Abstract

Cystic fibrosis (CF) is an autosomal recessive disorder characterized by the accumulation of sticky and heavy mucus that can damage several organs. CF shows variable expressivity in affected individuals, but it typically causes respiratory and digestive complications as well as congenital bilateral absence of the vas deferens in males. Individuals with classic CF usually have variants that produce a defective protein from both alleles of the *CFTR* gene. Individuals with other variants may present with classic, non-classic, or milder forms of CF due to lower levels of functional *CFTR* protein. This article reports the genetic analysis of a female with features of asthma and mild or non-classic CF. *CFTR* sequencing demonstrated that she is a carrier for a maternally derived 5T/12TG variant. Deletion/duplication analysis by multiplex ligation-dependent probe amplification (MLPA) showed the presence of an intragenic paternally derived duplication involving exons 7–11 of the *CFTR* gene. This duplication is predicted to result in the production of a truncated *CFTR* protein lacking the terminal part of the nucleotide-binding domain 1 (NBD1) and thus is likely to be a non-functioning allele. The combination of this large intragenic duplication and 5T/12TG is the probable cause of the mild or non-classic CF features in this individual.

Cystic fibrosis (CF [MIM 219700]) is an autosomal recessive disorder characterized by the accumulation of sticky and heavy mucus that can damage several organs leading to respiratory, digestive, and reproductive system problems. The disorder, depending on the variant type, can present with nearly 100% penetrance but shows variable expressivity in affected individuals, with symptoms including chronic cough, bronchiectasis, bacterial lung infections, lung and pancreatic fibrosis and insufficiency, poor growth and weight gain, fat soluble vitamin deficiency, malabsorptive stools, congenital absence of vas deferens (CAVD) and infertility in males, digital clubbing, and a relatively short life span due usually to pulmonary disease and/or liver failure^1^.

CF diagnosis is performed by a combination of clinical, laboratory, and genetic studies, usually starting with sweat chloride testing for symptomatic individuals. Affected individuals have higher amounts of chloride levels in their sweat due to an impaired ability to re-uptake chloride ions from their sweat. Abnormal sweat chloride levels are suggestive of CF for values equal to or higher than 60 mmol/L. Intermediate levels range from 40 to 59 mmol/L for 6-month or older individuals, or 30–59 mmol/L for individuals younger than 6 months of age. Genetic assessment should be performed for individuals with sweat chloride levels higher than 30 mmol/L. CF diagnosis is confirmed for symptomatic individuals with abnormal sweat chloride levels and harboring two pathogenic CF variants. Overall, CF patients demonstrate different clinical presentations ranging from classic CF to non-classic CF. Non-classic or mild CF is a term that is used to describe individuals manifesting with a milder form of CF compared to those with classic CF. These individuals usually do not present with pancreatic insufficiency, have intermediate sweat chloride levels, do not commonly show progressive lung function decline, and are usually diagnosed near adulthood^2^,^3^.

The incidence of CF is estimated to be approximately one in 2,500–3,500 for European Americans, one in 17,000 for African Americans, and one in 31,000 for Asian Americans^1^. Individuals with CF have pathogenic variants in both alleles of the cystic fibrosis transmembrane conductance regulator gene (*CFTR* [MIM 602421)]. The *CFTR* gene is located on chromosome 7q31.2, has 27 coding exons, and is the only gene known to be associated with *CFTR*-related disorders, including CF and CAVD. The *CFTR* gene encodes a transmembrane channel protein that transports chloride ions into and out of cells that produce mucus, sweat, saliva, tears, and digestive enzymes. *CFTR* variants that affect the function of the channel can cause extracellular mucus build-up; excessive thick and sticky mucus can then obstruct airways of lungs and ducts in the pancreas^4^.

The most common *CFTR* variants are single nucleotide substitutions and small deletions and duplications which may be easily detected by PCR-based techniques; however, large rearrangements have also been described^5^. The most common *CFTR* pathogenic variant in CF patients worldwide is a deletion of three nucleotides, c.1521_1523delCTT, which encodes part of the first nucleotide-binding domain (NBD1) of the *CFTR* protein. This in-frame deletion results in an abnormal *CFTR* protein that lacks the phenylalanine residue at position 508 (p.F508del)^5^,^6^,^7^,^8^. Individuals with other variants, including a CF disease-causing variant *in trans* with a short 5T nucleotide track and extended 12TG or 13TG di-nucleotide track in the intronic region chr7:117,188,661–117,188,689 of the *CFTR* gene, may present with classic, non-classic or milder forms of CF due to potentially lower levels of functional *CFTR* protein^9^,^10^,^11^,^12^,^13^,^14^,^15^,^16^,^17^,^18^.

In this report, we present the case of a female patient with chronic daily cough and sputum production, chronic sinusitis, mild reversible airflow obstruction, minimal bronchiectasis, and intermediate sweat chloride level, but no pancreatic insufficiency. Clinically, the patient was classified to have features of asthma and mild or non-classic CF. Gene sequencing and gene deletion/duplication analyses were performed and revealed the presence of a maternally derived 5T/12TG variant allele in addition to an intragenic paternally derived duplication involving exons 7–11 of the *CFTR* gene. This duplication is predicted to result in the production of a truncated *CFTR* protein lacking the terminal part of the NBD1 domain and thus is likely a loss of function (LOF) allele^7^. This finding suggest that the combination of this large intragenic duplication and the 5T/12TG variant is the probable cause of the mild or non-classic CF features in this individual.

## Results

### Genetic Analyses of Patient

**Figure 1:**
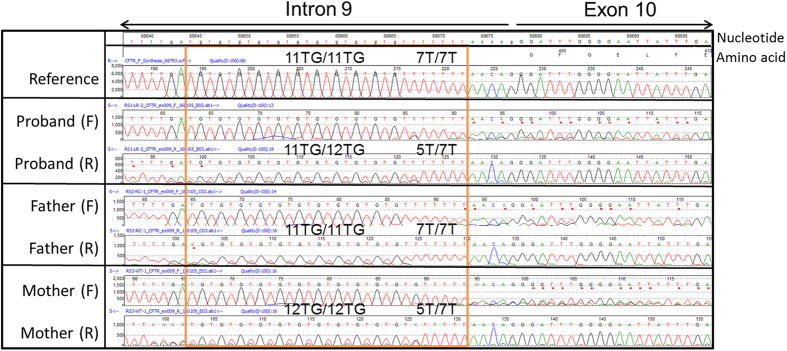
Sanger sequencing results for T/TG track. Reference DNA T/TG track (top electropherogram) on chr7:117,188,661-117,188,689 (GRCh37/hg19) is homozygous 7T; 11TG, or c.[1210−12[7];1210-34TG[11]]. Patient DNA (proband) T/TG track is heterozygous 5T/12TG; 7T/11TG (alleles overlap in figure), father is homozygous 7T/11TG, and mother is heterozygous 5T/12TG; 7TG/12TG. F, forward; R, reverse.

PCR followed by sequence analysis revealed a c.[1210−12[5];1210-34TG[12]] (5T/12TG) allele and a c.[1210−12[7];1210-34TG[11]] (7T/11TG) allele in the patient DNA (obtained from whole blood and buccal swab) (Fig. 1; see parental studies). No other variants were found in the *CFTR* gene.

**Figure 2:**
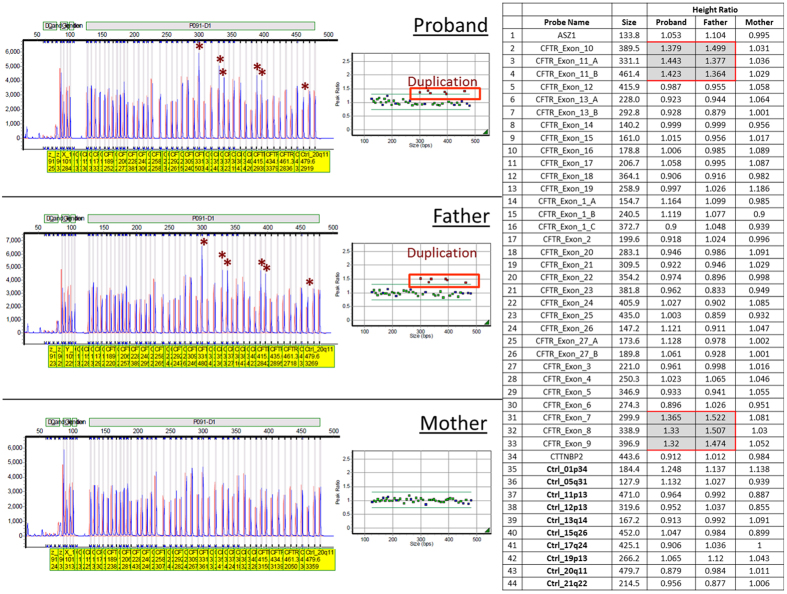
*CFTR* gene MLPA results. Proband and father (not mother) harbor a *CFTR* intragenic duplication involving exons 7, 8, 9, 10, 11 (minimal region of coordinates based on MLPA probe location is chr7:117,176,625-117,199,688-GRCh37/hg19). For each individual, the GeneMarker electrophoretograms are shown on the left and the normalized MLPA data is shown on the right. The table on the right lists the ratio of signal from each MLPA probe from each tested DNA in relation to the combined normal reference controls. A ratio of ~1.5 (test/control signal) is indicative of duplication (red squares). Asterisks are showing probes with higher ratio indicative of a duplication. The patient’s mother does not carry this duplication.

**Figure 3:**
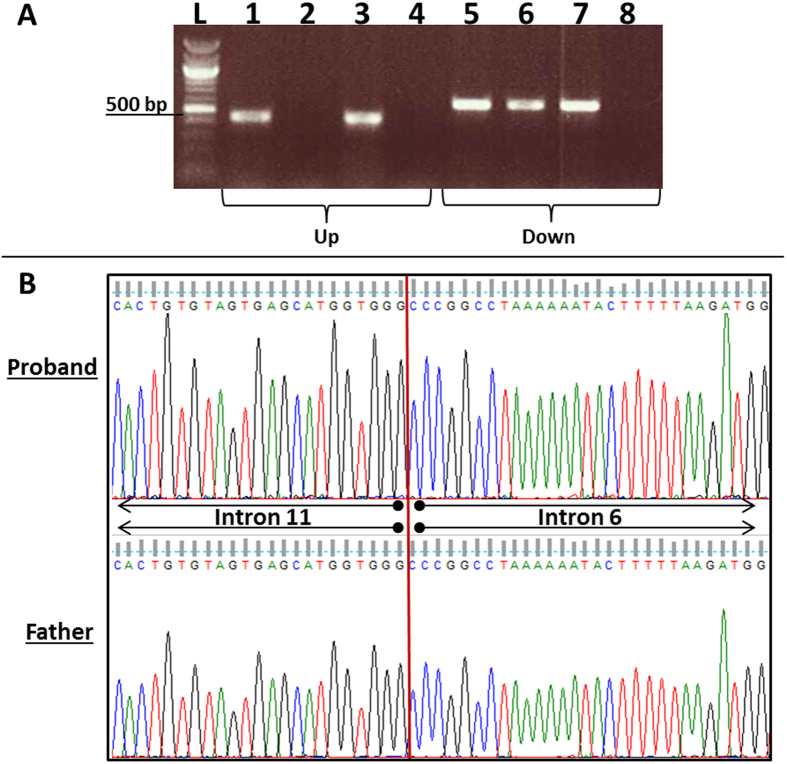
Patient and family PCR and Sanger sequencing results. (**A**) Only the two individuals harboring the duplication showed PCR amplification between introns 6 and 11 (upstream junction), while all samples showed PCR amplification in intron 11 (downstream junction), as previously reported^7^. L: ladder; upstream junction PCR products from 1: father, 2: mother, 3: proband, 4: blank (water); downstream junction PCR products from 5: father, 6: mother, 7: proband, 8: blank (water). (**B**) DNA sequencing electropherograms from the proband and her father show intron 11 and intron 6 sequences at the duplication breakpoint, as previously reported^7^.

Deletion/duplication analysis of the *CFTR* gene by multiplex ligation-dependent probe amplification (MLPA) revealed the presence of an intragenic duplication involving exons 7–11 of the *CFTR* gene (NM_000492.3), which include the minimal region of coordinates chr7:117,176,625–117,199,688 (based on MLPA probe location; GRCh37/hg19) (Fig. 2). MLPA results were confirmed using a breakpoint PCR designed by Hantash and colleagues to detect a recurrent duplication involving *CFTR* exons 7–11 (Fig. 3)^7^.

### Parental Studies

Parental buccal swabs were obtained to determine the *cis/trans* status of the two variants identified in our patient. Sanger sequencing and MLPA analysis were performed and revealed that the patient’s mother was a carrier for the 5T/12TG allele (Fig. 1) and the patient’s father was a carrier for the intragenic duplication involving exons 7–11 of the *CFTR* gene (Fig. 2). The parental MLPA results were further confirmed by breakpoint PCR amplification (Fig. 3).

## Discussion

In this study, we present the case of a female patient with features of asthma and mild or non-classic CF, who was referred for *CFTR* genetic analyses at our laboratory. The patient was found to have a 5T/12TG allele, *in trans* conformation with an intragenic duplication involving exons 7–11 of the *CFTR* gene. To our knowledge, this is the first CF patient described to have a *CFTR* intragenic duplication allele along with a 5T track allele. The most similar case previously reported was that of a Moroccan patient with congenital bilateral absence of the vas deferens (CBAVD [MIM 277180]) that had a *CFTR* heterozygous 5T/12TG in addition to a duplication involving exons 12–14^19^. No information regarding the *cis/trans* conformation of the two abnormalities was provided.

Previous studies have revealed that the length of the poly-T nucleotide track in intron 9 of the *CFTR* gene affects the levels of *CFTR* wild-type transcripts containing the adjacent exon (historically labeled exon 9, but current nomenclature listed as exon 10 in transcript NM_000492.3), with an individual with a heterozygous genotype consisting of a 5T allele and a 7T allele (such as the present case) having significantly reduced wild-type *CFTR* mRNA transcript (approximately 20% of all *CFTR* transcripts)^9^,^10^. Homozygosity for the 5T track has been previously reported in a male patient with non-classic CF presenting with sinopulmonary disease and infertility, but no history of pancreatitis. The patient genotype was homozygous 5T/11TG in the absence of other *CFTR* mutations after sequencing of all coding regions and splice sites^20^. Homozygosity for the 5T track has also been previously detected in a male patient with recurrent acute pancreatitis and a positive sweat test, but with normal respiratory function and urological phenotype. The patient was homozygous for 5T/12TG in the absence of 37 common European *CFTR* variants^21^. Overall, the 5T track, when found *in trans* conformation with a disease causing CF variant or in homozygosity, has been reported in individuals with non-classic CF, infertile males with CAVD, and unaffected individuals. The range of phenotype is believed to be related to the variable penetrance of the 5T track in causing abnormalities^9^,^22^. Furthermore, several studies have supported the idea that the length of the poly-TG nucleotide track, which immediately precedes the T track in intron 9 of *CFTR*, influences the penetrance of the 5T genotype; that is, it contributes to the increased levels of transcripts lacking exon 10. Specifically, individuals with an allele with a 12TG or 13TG repeat that is *in cis* with the 5T track (such as the present case) have been found to be more likely to show an abnormal clinical presentation than those individuals that had an allele with an 11TG repeat *in cis* with the 5T track^14^,^18^,^23^.

Other than the 5T track *in cis* with the 12TG track in a *CFTR* allele, our patient presented with an intragenic duplication involving exons 7–11. In order to assess how frequent *CFTR* duplications overlapping the one found in our patient are reported in publicly available data, non-CF databases (including the Coriell Cell Line Copy Number Variants, DECIPHER, and the Database of Genomic Variants) were queried but did not reveal any similar intragenic duplications in the *CFTR* gene, other than 10 subjects with a *CFTR* intragenic duplication involving exon 10 previously reported as pertaining to a pseudogene or segmental duplication sequence^24^,^25^,^26^,^27^,^28^,^29^,^30^,^31^. These data suggest that *CFTR* intragenic duplications involving multiple exons are not common, and further support that the duplication found in our patient is likely pathogenic. Furthermore, a large and public *CFTR* mutation database, Clinical and Functional Translation of *CFTR (CFTR*2)^5^, has described 16 alleles with the *CFTR* duplication involving exons 7–11 (reported in the database as *CFTR*dup6b-10), resulting in an allele frequency of 0.00011 (relative to a total of 141,341 mutations from 88,664 patients from 41 countries found in the database as of August 13^th^, 2015)^5^. The database reports the duplication as CF-causing with decreased lung function, association with pancreatic insufficiency, and average sweat chloride level of 106 mmol/L, but with a wide-range of severity when combined with a second CF-causing variant. The patient we describe in this manuscript had mild lung disease, no pancreatic insufficiency at the time of assessment, and an intermediate sweat chloride level. This milder phenotype in relation to the patients previously described is likely due to the combination of the duplication allele (predicted to result in a truncated *CFTR* protein lacking the terminal part of NBD1, see below) and the 5T/12TG allele, both of variable expressivity, and possibly other unknown modifiers^5^,^7^,^32^,^33^,^34^.

Other previously reported cases of CF patients that were found to have intragenic *CFTR* duplications that at least partially overlap exons 7–11 are relatively rare. These patients had classic *CFTR* mutations in addition to intragenic duplications, unlike our patient, who has a *CFTR* intragenic duplication in addition to a mild variant. As listed below, all these patients were described to have classic CF symptoms, with some reports indicating pancreatic disease and elevated sweat chloride levels. One report was that of a CF French male patient diagnosed at three months of age and who died at the age of 31, who had a clinical phenotype that included pancreatic insufficiency, severe lung disease, disseminated bronchiechtasis, and an elevated sweat chloride level of 90 mmol/L. He had a pathogenic p.G542X variant in addition to a maternally-derived duplication involving exons 4–9^32^. A second report was that of a classic CF Italian female patient with a clinical phenotype that included recurrent respiratory infections, pancreatic insufficiency, and a sweat chloride level above 100 mmol/L, who had a pathogenic maternally-derived p.F508del variant in addition to a paternally-derived duplication involving exons 7–18^33^. This duplication was predicted to result in a truncated CFTR protein. A third report was that of three duplications found from 233 CF chromosomes analyzed from classic CF patients, one involving exons 1–9, a second involving exons 4–11, and a third involving exons 7–11^34^. A fourth report was that of a classic CF Caucasian female patient with a clinical phenotype that included liver cirrhosis, pulmonary disease and a sweat chloride level of 110 mmol/L, who had a sister that reportedly died of CF. The patient was found to have a pathogenic maternally-derived p.F508del variant in addition to a paternally-derived duplication involving exons 7–11 (reported in the manuscript as dup6b-10) on a benign 7T/11TG haplotype^7^. Using DNA sequencing of the duplication junction fragment, the duplication breakpoint was found to be located in the intronic region surrounding the duplicated exons and was predicted to result in a truncated CFTR protein that lacked part of the NBD1 domain and was considered to be a null allele. The breakpoints were found within repetitive regions which were hypothesized to promote the duplication by a non-allelic homologous recombination mechanism. Two other unrelated CF Caucasian individuals obtained from another laboratory were reported in the same study, each having a pathogenic *CFTR* variant in addition to the exon 7–11 duplication with the same duplication breakpoint, thus suggesting there is likely a founder effect for this duplication^7^. We performed the breakpoint PCR using the primers provided in the aforementioned study and confirmed the same breakpoints flanking the duplication in both our patient and her father.

Our genetic analyses suggest that the presence of two mild variants, the 5T/12TG variant *in trans* with the exon 7–11 duplication allele found in the current case, may result in a mild/non-classic CF presentation^20^,^21^. Interestingly, previous studies have suggested that expression of only 8% of wild-type *CFTR* mRNA transcripts (containing exon 10) from bronchial epithelial cells may be necessary to maintain a normal lung phenotype^35^. Future gene expression studies may be useful to determine the amount of functional CFTR protein produced in patients harboring a combination of unusual or mild variants, such as the ones described in our patient. These studies may increase our understanding of phenotype/genotype correlations in CF and help the research community better define the threshold of wild-type transcripts required for an unaffected CF status. This information will then be useful for the development of therapeutic options, for example, using gene therapy strategies.

This case illustrates the importance of searching for variations in the T and TG nucleotide tracks in intron 9, as well as large intragenic rearrangements within the *CFTR* gene in patients with non-classic mild and possibly classic CF disease who have an absence of the most commonly found variants (single nucleotide substitutions and small indels) in one or both *CFTR* alleles.

## Methods

### DNA extraction

Patient whole blood collected in purple-top tube was received at the Indiana University Molecular Genetics Diagnostic Laboratory, Indianapolis, IN, and DNA extracted using the Qiagen’s Gentra Puregene Blood Kit (Qiagen, Germantown, MD) following the manufacturer’s instructions. Parental and patient buccal swabs were collected and received at the Indiana University Molecular Genetics Diagnostic Laboratory, Indianapolis, IN, and DNA was extracted using the Qiagen’s Gentra Puregene Blood Kit (Qiagen, Germantown, MD) following the manufacturer’s instructions.

### Patient clinical information

Informed consent for molecular genetics testing was obtained from the patient and her parents. The patient was a 27-year-old female of normal body weight with a history of chronic daily cough, sputum production, chronic sinusitis, recurrent lower respiratory tract infections, and intermediate sweat chloride levels (58 mmol/L). The patient had no pancreatic insufficiency, no history of malabsorptive stools, and no digital clubbing. She was found to have no evidence of wheezing or crackles on lung examination. Her pulmonary function tests demonstrated mild airflow obstruction, while high resolution chest imaging showed minimal bronchiectasis. Bronchodilator reactivity was not assessed. She was clinically diagnosed to have mild/non-classic CF with features of asthma. The patient had no family history of CF or other respiratory illnesses.

All methods were carried out in accordance with guidelines and regulations of the Molecular Genetics Diagnostic Laboratory at Indiana University. An IRB protocol (protocol # 1401263105) was approved by the Human Subjects Office at Indiana University.

### PCR and Big Dye Sanger Sequencing

Patient DNA was tested for *CFTR* coding and splicing variants in all 27 coding exons, while parental DNA was tested for intron 9T/TG track. Polymerase chain reaction (PCR) was performed using HotStar Taq DNA Polymerase kit (Qiagen, Germantown, MD). Briefly, 50 ng of genomic DNA was amplified in a 25 μL reaction that contained 1 μM of each M13-tailed primer (Integrated DNA Technologies, Inc., Coralville, IA; primer sequences are available upon request), 0.2 mM GeneAmp dNTP blend (Life Technologies, Carlsbad, CA), 1 mM MgCl^2+^, 1 unit of HotstarTaq DNA polymerase, and 10x buffer with 15 mM MgCl^2+^. Touchdown PCR was performed using the following reaction conditions: 95 °C for 15 min; 14 cycles of 94 °C for 45 sec, 65 °C for 30 sec with a 0.5 °C decrease per cycle, and 72 °C for 1 min; 24 cycles of 94 °C for 45 sec, 58 °C for 30 sec, and 72 °C for 1 min; 72 °C for 10 min. PCR products were analyzed using agarose gel electrophoresis to confirm appropriate amplification. Products were then sequenced by automatic fluorescent DNA sequencing using an Applied Biosystems (ABI) Prism 3130*xl* or 3500*xl* Genetic Analyzer in conjunction with the ABI BigDye Terminator v3.1 cycle sequencing kit chemistry and protocol (ABI, Foster City, CA). Sequences were analyzed using Mutation Surveyor software V4.0.7 (SoftGenetics, State College, PA) using the *CFTR* transcript NM_000492.3 as reference.

Breakpoint PCR was performed on patient and parental DNA using previously published primer sequences^7^. Briefly, primers Dup6b10upF (5′-TGTAAAACGACGGCCAGTCAGCATAAGATCCTGAAGGTTTG-3′) and Dup-6b10upR (5′-CAGGAAACAGCTATGACCAACACAAAGTAACTAAGGCTCTGGT-3′) were used to detect the upstream junction fragment, while primers Dup6b10dnF (5′-TGTAAAACGACGGCCAGTTGGCAATGGGGTTGGGAAGT-3′) and Dup6b10dnR (5′-CAGGAAACAGCTATGACCCTGCTCCTCACTATCACAGTCAGTGA-3′) were used to detect the downstream junction fragment. PCR, electrophoresis, and sequencing conditions were identical to the ones described above. Sequences were analyzed using Mutation Surveyor software V4.0.7, FinchTV software V1.4.0 (Geospiza Inc., Seattle, WA), and the UCSC genome browser^26^ using the *CFTR* transcript NM_000492.3 as reference.

### Multiplex Ligation-Dependent Probe Amplification (MLPA)

Patient DNA was tested for *CFTR* exonic deletions and duplications by MLPA following the manufacturer’s instructions for the MLPA P091-D1 *CFTR* probemix (MRC-Holland, Amsterdam, Netherlands). This pre-design kit contains probes for all coding exons (1–27) of the *CFTR* gene according to transcript NM_000492.3. Analysis was performed using SoftGenetics Gene Marker software v2.6.0 using three normal samples (containing no deletions or duplications within the region tested) as references. The normal range for a test sample (no deletion/duplication) is expected to have a peak height ratio from 0.8–1.2. The range consistent with a heterozygous deletion for a test sample is expected to have a peak height ratio from 0.4–0.6. The range consistent with a heterozygous duplication for a test sample is expected to have a peak height ratio from 1.4–2.0.

## Additional Information

**How to cite this article**: Celestino-Soper, P. B. S. *et al*. Intragenic *CFTR* Duplication and 5T/12TG Variant in a Patient with Non-Classic Cystic Fibrosis. *Sci. Rep.* **6**, 38776; doi: 10.1038/srep38776 (2016).

**Publisher's note:** Springer Nature remains neutral with regard to jurisdictional claims in published maps and institutional affiliations.
